# Supplementary material for: Chromatin remodeling of histone H3 variants underlies epigenetic inheritance of DNA methylation
Source: bioRxiv. 2023 Aug 2:2023.07.11.548598. Originally published 2023 Jul 11. Preprint. [Version 2] doi: 10.1101/2023.07.11.548598 (PMC10369972; doi:10.1101/2023.07.11.548598)
Supplement: Supplement 1 [file media-1.pdf]

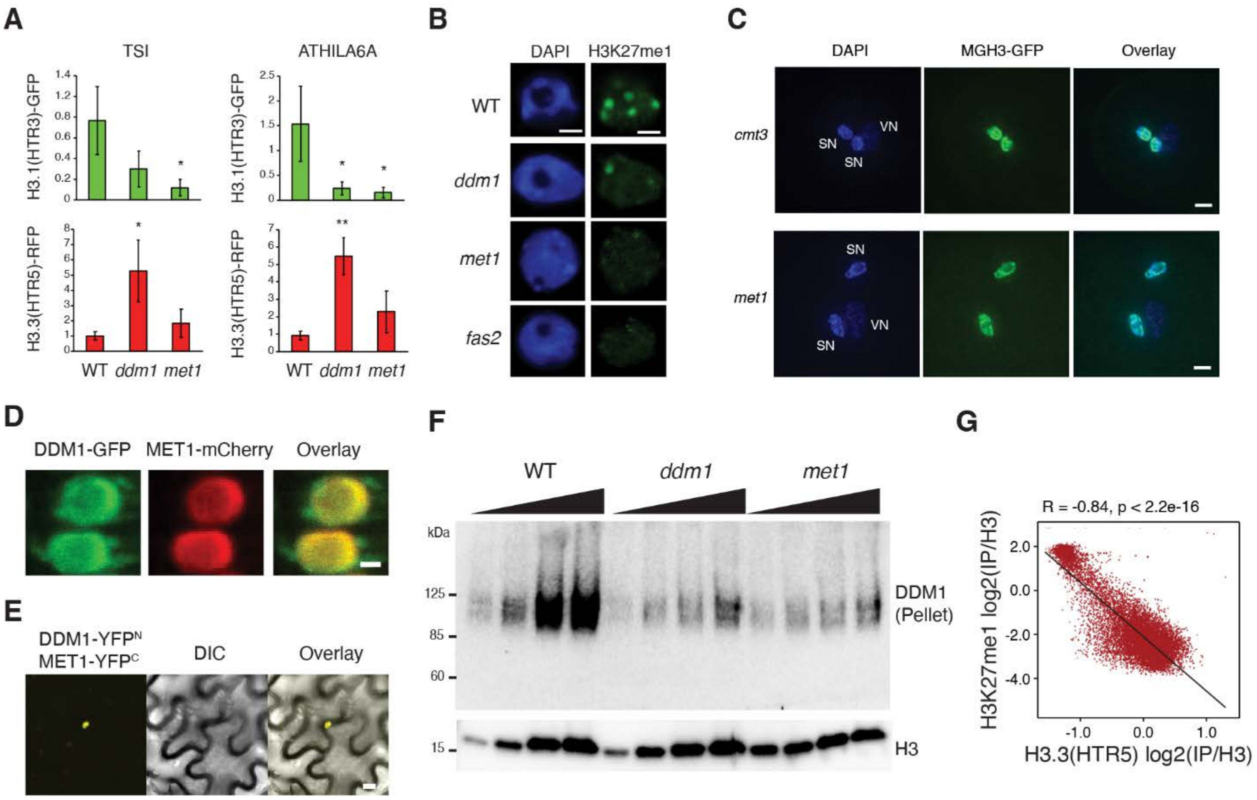

Supplementary Figure S1. MET1 participates in histone remodeling by DDM1. (A) ChIP-qPCR amplification of *TSI* (*ATHILA*) and *ATHILA6A* repeats in wild-type (WT), *ddm1*, and *met1* with 10-d-old seedling tissues. ChIP signals of H3.1(HTR3)-GFP and H3.3(HTR5)-RFP were normalized to H3. Error bars indicate standard deviations (biological replicates; n=3). P values of statistical difference with WT are shown above each mutant (one-way Anova adjusted with Tukey's Honest Significant Difference method; * p-value<0.05, ** p-value<0.01). (B) Immunofluorescence of H3.1-associated histone modification H3K27me1 in 3-week-old leaves of WT, *ddm1*, *met1*, and *fas2* (*caf-1*). DAPI was used for DNA staining. Scale bars indicate 2 µm. (C) Male Germline-specific Histone H3.3 MGH3-GFP localization in sperm nuclei of Arabidopsis pollen grains. DAPI staining was used to visualize vegetative (VN) and sperm nuclei (SN). Mislocalization to the nuclear periphery was observed in *met1* mutants, but not in *cmt3*. Scale bars indicate 2 µm. (D) Co-localization of DDM1-GFP and MET1-mCherry in the nucleus. Scale bar indicates 2 µm. (E) Bimolecular fluorescence complementation using DDM1 fused with N-terminal YFP (YFPN) and MET1 with C-terminal YFP (YFPC). Scale bar indicates 5 µm. Complementation is defined by the yellow nucleus. (F) Western blot analysis of endogenous DDM1 from the chromatin/pellet (P) fraction of WT, *ddm1*, and *met1* backgrounds. Anti-H3 was used as loading control. Serial dilutions (1:2) were made for each sample (gradient) indicating that both *ddm1* and *met1* mutants had between ½ and ¼ WT levels of chromatin-bound DDM1. (G) Genome-wide negative correlation between H3K27me1 (H3.1) and H3.3(HTR5)-RFP ChIP-seq in wild-type (Figure 1D). P and R values indicate statistical significance and Pearson correlation coefficients, respectively.

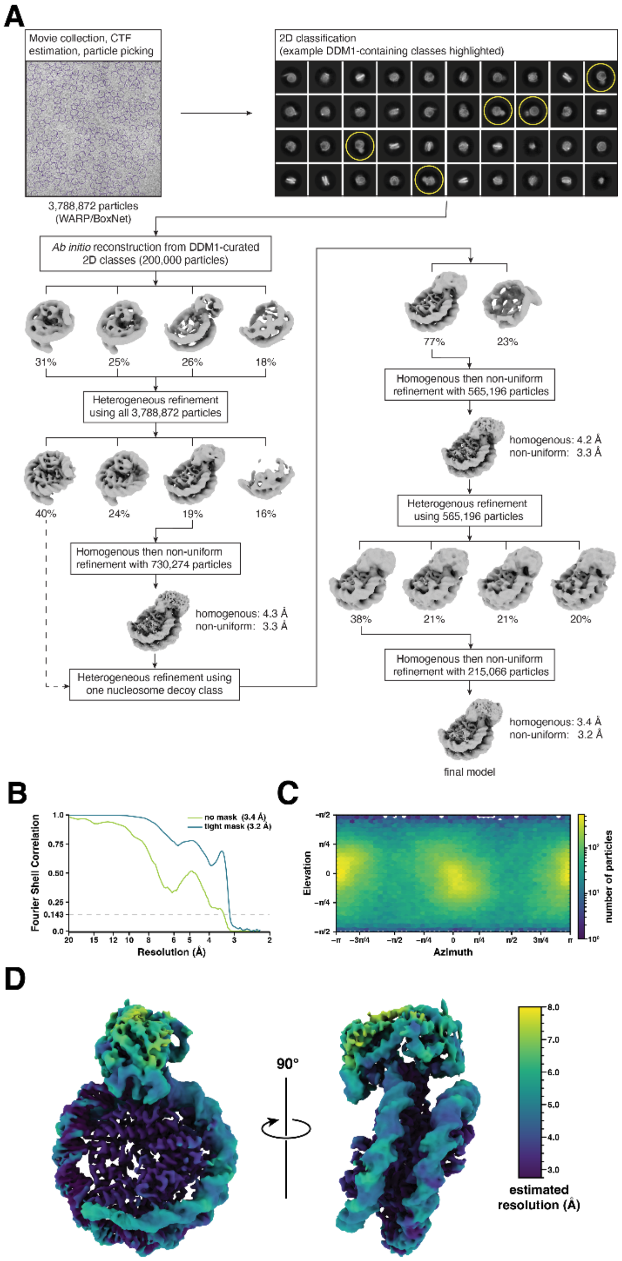

Supplementary Figure S2. Cryo-EM data processing workflow and reconstruction metrics. (A) Following cryo-EM movie collection, motion correction, averaging, CTF estimation, and particle picking were performed using WARP [96]. Example particle picks are shown as purple circles (top left). Particles were then imported into cryoSPARC [97] for 2D classification as well as 3D classification and refinement. Examples of DDM1-containing 2D classes, which were used to generate the ab initio models are highlighted with yellow circles (top right). Class distributions are indicated for each heterogenous refinement step. Reconstruction resolutions after homogenous and non-uniform refinement are indicated next to the corresponding models. (B) Fourier Shell Correlation (FSC) plots of the DDM1-nucleosome reconstruction using no mask (green) and a tight mask (blue). Resolution values at FSC 0.143 are indicated. (C) Angular distribution plot of reconstruction projections. The heat map indicates the number of particles per viewing angle. (D) The DDM1-nucleosome complex reconstruction, colored by estimated local resolution from cryoSPARC.

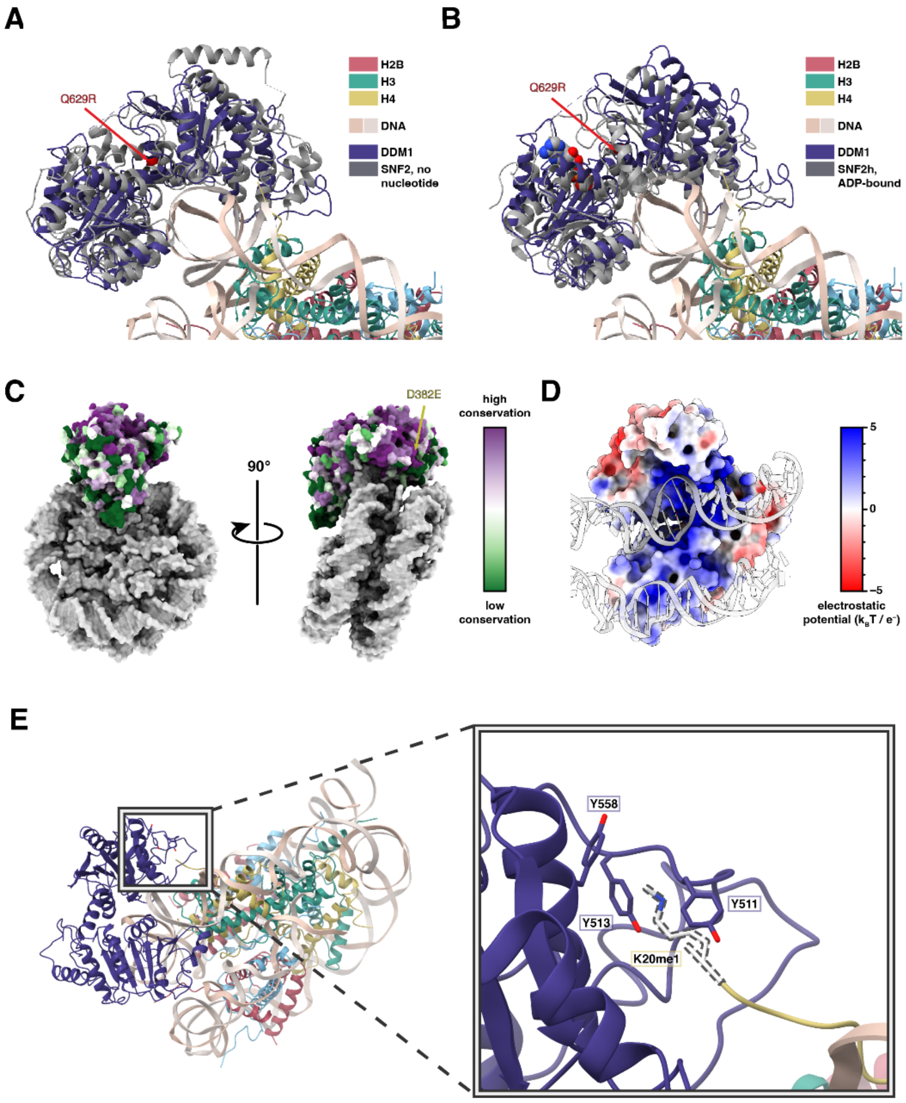

Supplementary Figure S3. Structural comparison of DDM1 with Snf2 family remodelers. The structures of (A) Snf2-bound nucleosomes in the absence of nucleotide (PDB code 5X0Y) and (B) Snf2h in the presence of ADP (PDB code 6NE3) (red) superimposed on the structure of DDM1 bound to nucleosome. The Q629R mutation in DDM1 is shown with a red arrow. Alignment was performed using only the nucleosome core particle for each structure. In the presence of bound ADP, the two domains appear in a more closed conformation than the nucleotide free state. The DDM1/nucleosome complex that was reconstructed represents the nucleotide free state. Note that the sample used for the Snf2h structure was prepared with ADP-BeF_3 but only ADP was observed in the density. (C) Surface representation of the DDM1-bound nucleosome colored according to degree of DDM1 conservation. Conservation scores were calculated using the Consurf server [98] among twenty manually-curated and highly related sequences—such as LSH and HELLS—aligned using Clustal Omega [99]. The D382A mutation in DDM1 is indicated with a yellow arrow. (D) The electrostatic potential of DDM1 (colored surface) displays a positively-charged groove along the DNA (grey cartoon) interface. Electrostatic surface calculations were performed by APBS [100] with a solvent ion concentration of 0.15 M at 298 K using the PARSE force field. (E) The tail of histone H4 extends toward DDM1 such that the residue K20 would be within striking distance of three aromatic residues in DDM1 forming an aromatic cage. The inset indicates a modeled mono-methylated lysine residue with a dashed outline.

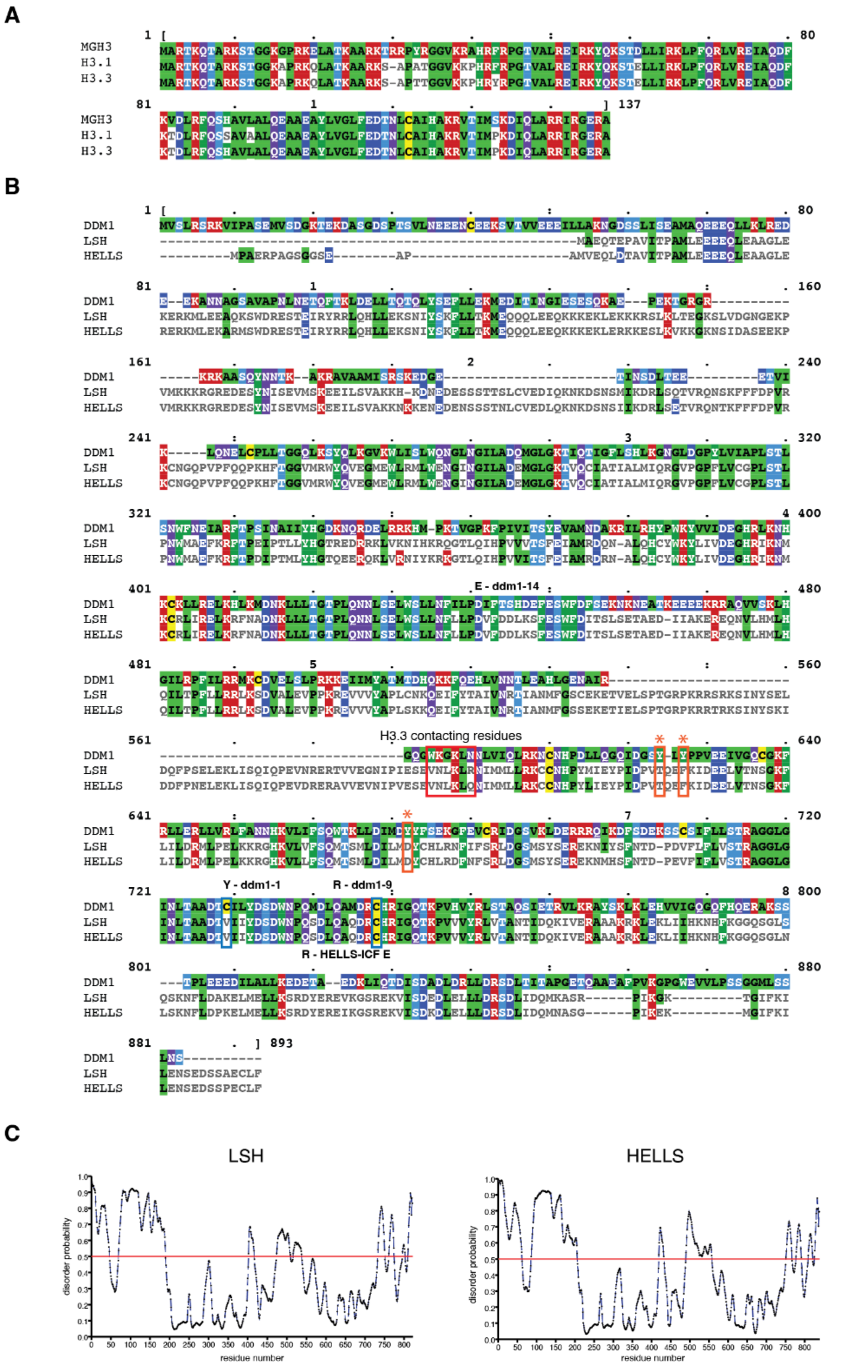

Supplementary Figure S4. Amino acid sequence alignments. (A) The sequence alignment of histone MGH3, H3.1, and H3.3 generated with MView [101]. (B) The sequence alignment of DDM1, LSH, and HELLS. H3.3 contacting residues (WKGKLN) of DDM1 are indicated with a red bar. Tyrosine residues Y511, Y513, and Y558 (DDM1 aromatic cage) are indicated with orange asterisks. Cysteine residues C615 and C634 (DDM1 disulfide bond) are indicated with blue asterisks. Three DDM1 hypomethylation mutations (*ddm1-1*, *ddm1-9* and *ddm1-14*) and one HELLS mutation (ICF proband family E) are indicated by substituted residues above and below the mutated location, respectively. Compared to DDM1, LSH has 90.6% coverage with 34.8% identity while HELLS has 92.8% coverage with 33.8% identity. (C) Intrinsically disordered regions of LSH and HELLS using PrDOS [102].

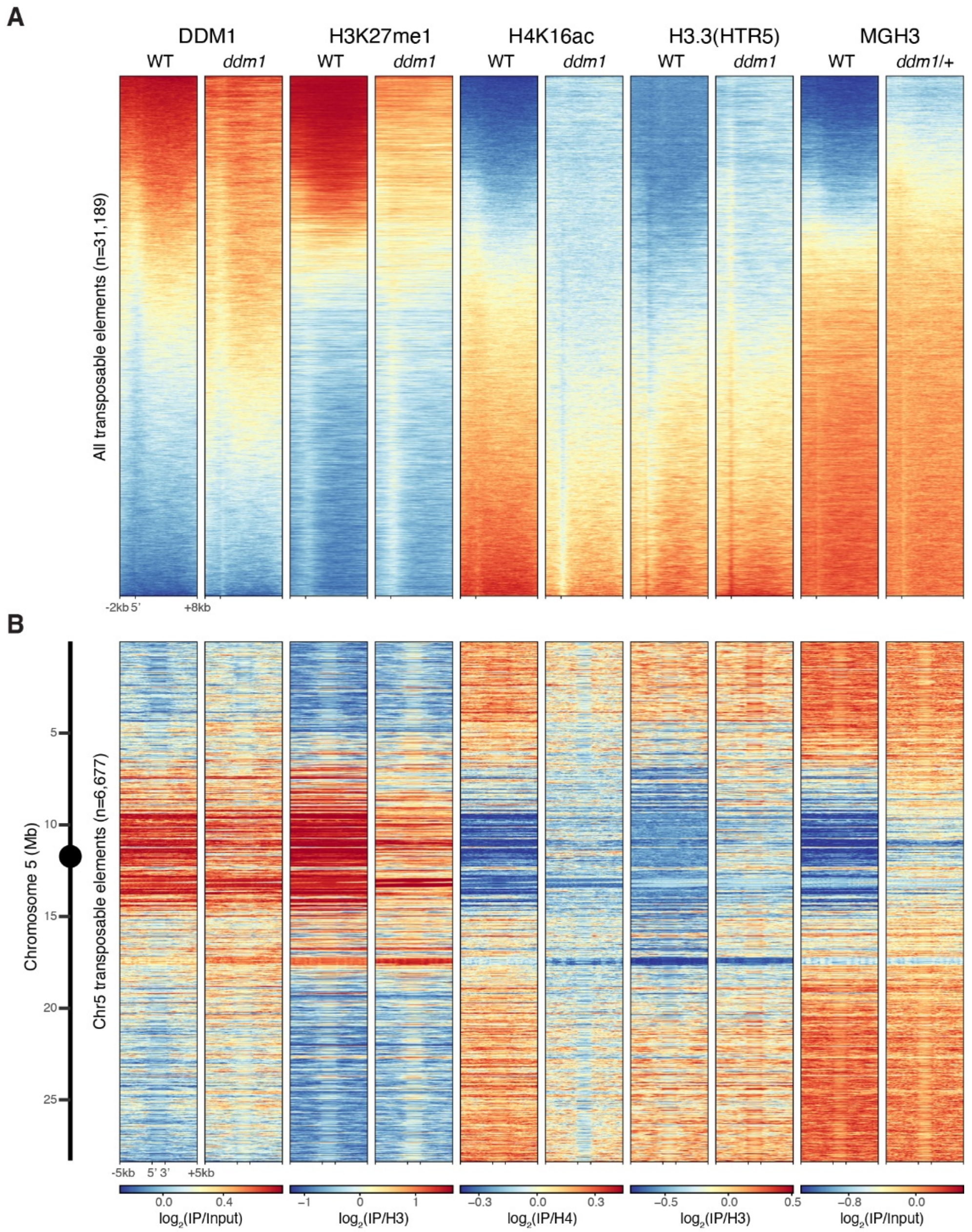

Supplementary Figure S5. ChIP-seq data for all transposable elements in WT and *ddm1*. (A) Heatmaps of DDM1, H3K27me1, H4K16ac, H3.3(HTR5) ChIP-seq of wild-type (WT) and *ddm1* genotypes, as well as MGH3 in pollen from WT and *ddm1*/+ plants, for all transposable elements annotated in TAIR10. Heatmaps were generated using Deeptools [95], where all 31,189 TEs were aligned by their 5' end with 2kb upstream and 8kb downstream with a binsize of 10bp, and sorted based on DDM1 levels in WT. (B) Similar heatmaps were generated using Deeptools, where the 6,677 TEs located on chromosome 5 were scaled to 2kb, represented with 5kb upstream and 5kb downstream with a binsize of 10bp. TEs were kept in order of their location on the chromosome, shown by the scale on the left hand-side. This view highlights that DDM1 preferentially targets peri-centromeric TEs in WT. Both heatmaps highlight correlation between DDM1 and H3K27me1 and anti-correlation with H4K16ac, H3.3 and MGH3 levels in both genotypes, as well as the loss of DDM1 and H3K27me1 from peri-centromeric TEs in *ddm1* accompanied by an increase in H4K16ac, H3.3 and MGH3.

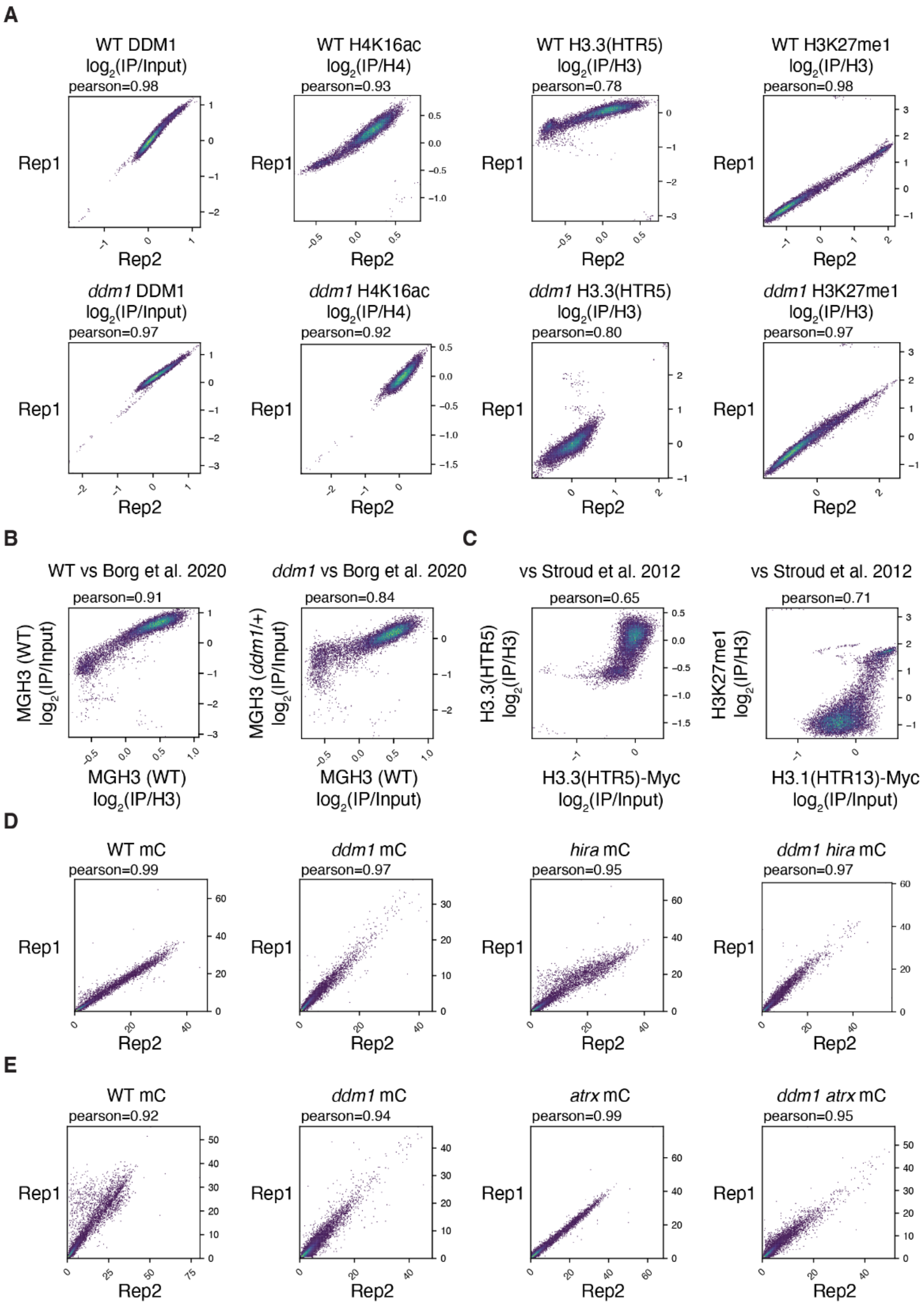

Supplementary Figure S6. Correlations between ChIP-seq replicates and between WGBS replicates. (A) Comparisons of DDM1, H4K16ac, H3.3(HTR5)-RFP and H3K27me1 ChIP-seq data between replicates of each genotype. Pearson correlations are shown. (B) Comparisons of MGH3 in WT and ddm1/+ pollen with previously published MGH3 ChIP-seq [47]. (C) Comparisons of H3.3(HTR5)-RFP and H3K27me1 ChIP-seq in WT with previously published H3.3(HTR5)-Myc and H3.1(HTR13)-Myc, respectively [40]. (A-C) Each replicated IP has been normalized to its respective input. (D, E) Comparisons of DNA methylation levels in each replicate for all genotypes grown and processed at the same time for group A (D) and group B (E), respectively.

Supplementary Video S1 - Live imaging of DDM1-GFP and H3.3(HTR5)-RFP during transition from M phase to G1 phase.

Supplementary Video S2 - Live imaging of DDM1-GFP and H3.3(HTR5)-RFP during transition from G2 phase to M phase.

Supplementary Table S1 - Coordinates of hypermethylated differentially methylated regions in ddm1 hira vs ddm1, Related to Figure 2 (.xlsx).

Supplementary Table S2 - Coordinates of stable and revertant DMRs in ddm1 identified in Colomé-Tatché et al., 2012, and random control regions, Related to Figure 6 (.xlsx).

Supplementary Table S3. List of mutants in this study, Related to STAR Methods

Mutants	Mution type	Notes
ddm1-2	EMS mutant (nucleotide: G to A)	Hypomorphic; the splicing defect leads to a deletion, a frameshift and premature translation termination; DNA hypomethylation
ddm1-10	T-DNA (SALK_093009)	T-DNA insertion into exon region; used for crossing with MGH3-GFP
met1-1	EMS mutant (amino acid: P 1300 S)	Hypomorphic; amino acid substitution in catalytic domain; DNA hypomethylation; delayed flowering
met1-7	T-DNA (SALK_076522)	T-DNA insertion into exon region; used for crossing with MGH3-GFP
cmt3-11	EMS mutant	Single nucleotide substitution leading to a nonsense mutation; used for crossing with MGH3-GFP
fas2-4	T-DNA (SALK_033228)	Null; no transcript detectable; fasciation
hira-1	WiscDsLox362H05	Reduced fertility
atrx-2	SAIL_861_B04	Reduced fertility

Supplementary Table S4. Primer sequences, Related to STAR Methods

Cloning primers

Name	Primer sequence (5' to 3')	Locus	Note
DDM1_attB1	GGGGACAAGTTTGTACAAAAAAGCAGGCT ATTACTAATTGTGTCGACAAATCC	AT5G66750	for cloning into pDONR221
DDM1_attB2	GGGGACCACTTTGTACAAGAAAGCTGGGT AATCCCAAATCCAAAACATAAGATC	AT5G66750	for cloning into pDONR221
MET1_attB1	GGGGACAAGTTTGTACAAAAAAGCAGGCT TCATGGTAAAATGTTAGTTCTCG	AT5G49160	for cloning into pDONR221
MET1_attB2	GGGGACCACTTTGTACAAGAAAGCTGGGT CTGGACAAACTTTATTTCGAC	AT5G49160	for cloning into pDONR221

Genotyping

Name	Primer sequence (5' to 3')	Locus	Note
ddm1-2 CAPS F	GTTGGACAGTGTGGTAAATTCCGCT	AT5G66750	RsaI digestion
ddm1-2 CAPS R	GAGCTACGAGCCATGGGTTTGTGAAACGTA	AT5G66750	RsaI digestion
ddm1-10 F	GCAAGCCATGGACAGATGCCACAG	AT5G66750	
ddm1-10 R	CAGAGGGCCAATTGTTTTCATCAC	AT5G66750	
met1-1 F	CTCTTTAGTAGAAGTTGGCATG	AT5G49160	HaeIII digestion
met1-1 R	ATATGTATGTATAGATATTTTCTCC	AT5G49160	HaeIII digestion
hira-1 LB	CTACTAAAATTTGAGGCCGGG	AT3G44530	
hira-1 RB	GAGAGTCACTGTTTTGGCTGG	AT3G44530	
atrx-2 LB	AGGAACCCTCACAGCTTCTTC	AT1G08600	
atrx-2 RB	TCACATGGATGGCTTCTTTTC	AT1G08600	
HTR3-GFP-F	TAGTGCAGTCGCAGCTCTTC	AT3G27360	
HTR3-GFP-R	TTTGTACAAGAAAGCTGGGTCAG	AT3G27360	

ChIP-qPCR

Name	Primer sequence (5' to 3')	Locus	Note
TSI F	ATCCAGTCCGAAGAACGCGAACTA		
TSI R	TCACTTGTGAGTGTTCGTGAGGTC		
ATHILA6A F	ACAGGAAGTGGGCGCACACC	AT5G32511	
ATHILA6A R	CTCACAACGACGCAAGTGATCT	AT5G32511	

ATPase assay substrates

Name	Primer sequence (5' to 3')	Locus	Note
Widom601 0N60 F	AGAGTGGGAGCTCGGAACACTATCCGAC		
Widom601 0N60 R	CTGGAGAATCCCGGTGCC		

Supplementary Table S5. ChIP-sequencing libraries metrics, Related to STAR Methods

Genotype	Sample	IP or Control	Replicate	Total reads	All mapped reads	(% total)	Uniquely mapped reads	(% total)
WT	DDM1	IP	Rep1	40,515,724	37,212,763	91.85%	21,468,053	52.99%
WT	DDM1	Input	Rep1	47,290,771	45,973,121	97.21%	32,105,406	67.89%
WT	DDM1	IP	Rep2	34,645,829	32,569,880	94.01%	19,276,714	55.64%
WT	DDM1	Input	Rep2	51,279,688	49,817,225	97.15%	34,561,985	67.40%
ddm1	DDM1	IP	Rep1	50,102,086	47,183,497	94.17%	27,125,034	54.14%
ddm1	DDM1	Input	Rep1	39,982,483	39,028,473	97.61%	26,984,304	67.49%
ddm1	DDM1	IP	Rep2	49,577,213	43,872,422	88.49%	24,804,809	50.03%
ddm1	DDM1	Input	Rep2	33,380,544	32,612,138	97.70%	22,752,619	68.16%
WT	H3K27me1	IP	Rep1	30,078,663	29,656,796	98.60%	11,522,922	38.31%
WT	H3K27me1	H3	Rep1	22,622,734	22,483,854	99.39%	16,142,362	71.35%
WT	H3K27me1	IP	Rep2	50,994,633	40,503,003	79.43%	13,950,016	27.36%
WT	H3K27me1	H3	Rep2	60,442,954	59,862,967	99.04%	45,680,564	75.58%
ddm1	H3K27me1	IP	Rep1	26,031,998	25,885,357	99.44%	18,435,013	70.81%
ddm1	H3K27me1	H3	Rep1	28,806,975	28,258,079	98.09%	10,926,824	37.93%
ddm1	H3K27me1	IP	Rep2	37,494,713	28,836,811	76.91%	9,554,115	25.48%
ddm1	H3K27me1	H3	Rep2	52,356,409	51,800,730	98.94%	38,115,613	72.80%
WT	H4K16ac	IP	Rep1	29,867,512	29,113,254	97.47%	19,477,351	65.21%
WT	H4K16ac	H4	Rep1	32,025,461	31,151,067	97.27%	17,149,656	53.55%
WT	H4K16ac	IP	Rep2	49,958,674	48,846,091	97.77%	33,687,425	67.43%
WT	H4K16ac	H4	Rep2	49,482,528	48,239,575	97.49%	26,621,503	53.80%
ddm1	H4K16ac	IP	Rep1	44,578,514	43,566,178	97.73%	28,553,612	64.05%
ddm1	H4K16ac	H4	Rep1	59,698,668	20,431,979	34.23%	12,114,825	20.29%
ddm1	H4K16ac	IP	Rep2	40,697,481	39,804,154	97.81%	26,494,041	65.10%
ddm1	H4K16ac	H4	Rep2	33,698,770	32,821,242	97.40%	17,607,160	52.25%
WT	HTR5	IP	Rep1	89,816,986	89,262,349	99.38%	74,274,600	82.70%
WT	HTR5	H3	Rep1	43,362,049	42,608,947	98.26%	33,866,720	78.10%
WT	HTR5	IP	Rep2	47,569,522	47,259,571	99.35%	40,618,380	85.39%
WT	HTR5	H3	Rep2	62,187,338	61,578,996	99.02%	46,846,643	75.33%
ddm1	HTR5	IP	Rep1	91,478,171	90,971,481	99.45%	68,533,360	74.92%
ddm1	HTR5	H3	Rep1	43,901,988	43,551,892	99.20%	31,424,017	71.58%
ddm1	HTR5	IP	Rep2	88,251,305	87,728,045	99.41%	67,051,725	75.98%
ddm1	HTR5	H3	Rep2	77,052,248	76,533,910	99.33%	51,185,737	66.43%
WT	MGH3	IP	Rep1	114,691,469	110,674,811	96.50%	97,134,182	84.69%
WT	MGH3	Input	Rep1	42,239,090	39,899,471	94.46%	25,189,691	59.64%
ddm1 /+	MGH3	IP	Rep1	28,958,163	23,043,589	79.58%	17,882,624	61.75%
ddm1 /+	MGH3	Input	Rep1	25,645,477	24,623,469	96.01%	14,422,150	56.24%

Supplementary Table S6. Bisulfite sequencing libraries metrics, Related to STAR Methods

Genotype	Group	Replicate	Total raw reads	All mapped reads	(% total)	unique alignments	(% total)	deduplicated	(% unique)	Cytosine covered	Average coverage	Non conversion rate (% mC/C in Pt)
WT	A	Rep1	32,230,036	30,942,651	96.01%	24,745,052	76.78%	11,017,686	44.52%	90.41%	8.17	0.269861
WT	A	Rep2	53,613,049	52,389,181	97.72%	42,053,916	78.44%	9,566,292	22.75%	88.18%	6.78	0.51281
hira	A	Rep1	17,362,101	16,566,981	95.42%	12,957,483	74.63%	5,285,057	40.79%	77.90%	3.93	0.270725
hira	A	Rep2	44,991,888	42,569,624	94.62%	33,798,990	75.12%	4,608,590	13.64%	78.21%	3.22	0.706732
ddm1hira	A	Rep1	21,758,809	17,461,284	80.25%	13,837,031	63.59%	5,419,437	39.17%	77.38%	3.94	0.287453
ddm1hira	A	Rep2	48,425,900	36,805,003	76.00%	28,725,572	59.32%	8,623,617	30.02%	84.11%	6.16	0.548739
ddm1	A	Rep1	27,502,435	26,294,284	95.61%	20,404,561	74.19%	7,057,712	34.59%	83.48%	5.30	0.266461
ddm1	A	Rep2	37,878,589	37,470,061	98.92%	29,204,434	77.10%	9,163,719	31.38%	83.05%	6.49	0.537523
WT	B	Rep1	30,561,004	24,364,737	79.72%	18,151,083	59.39%	9,660,873	53.22%	77.57%	7.07	0.589116
WT	B	Rep2	34,550,917	30,417,035	88.04%	22,452,116	64.98%	13,366,408	59.53%	79.20%	9.83	0.587723
ddm1atrx	B	Rep1	23,525,857	19,197,760	81.60%	14,136,528	60.09%	8,880,690	62.82%	71.88%	6.43	0.595414
ddm1atrx	B	Rep2	37,170,647	35,040,723	94.27%	24,906,077	67.00%	14,078,236	56.53%	79.15%	10.33	0.667431
ddm1	B	Rep1	36,520,861	35,544,986	97.33%	26,379,181	72.23%	10,534,593	39.94%	78.73%	7.54	0.621665
ddm1	B	Rep2	20,358,084	19,257,875	94.60%	14,528,944	71.37%	6,440,673	44.33%	73.49%	4.70	0.923219
atrx	B	Rep1	39,661,456	39,246,987	98.95%	29,154,984	73.51%	7,359,080	25.24%	79.63%	5.21	0.845968
atrx	B	Rep2	38,694,008	38,125,949	98.53%	28,077,230	72.56%	9,110,947	32.45%	79.89%	6.57	0.661121

Supplementary Table S7. Cryo-EM data collection and reconstruction statistics for the DDM1-nucleosome complex, Related to STAR Methods

**Data collection**	
Microscope	Titan Krios
Voltage (keV)	300
Magnification	81,000×
Defocus range (μm)	−1.0 to −2.2
Detector	K3
Pixel Size (Å)	1.1
Total exposure ($e^-/Å^2$)	71.2
Exposure rate ($e^-/Å^2$/sec)	14.8
Exposure per frame ($e^-/Å^2$)	2.37
Micrographs collected	8,165

**Initial processing**	
Micrographs used	7,811
Initial particles	3,788,872

**Reconstruction**	
Final particles	215,066
Symmetry	C1
Map sharpening B-factor ($Å^2$)	57.8
Half maps resolution (unmasked / masked)	
FSC 0.143	3.4 / 3.2

Supplementary Table S8. Model refinement and validation statistics for the DDM1-nucleosome complex, Related to STAR Methods

Statistics are provided for the full model as well as the individual octamer, DNA and DDM1 components.

**Refinement**	**Full model**	**Octamer**	**DNA**	**DDM1**
Protein residues	1217	749	——	468
Nucleic acid residues	282	——	282	——
Model resolution (unmasked / masked)				
FSC 0.5	3.2 / 3.2			
FSC 0.143	2.8 / 2.8			
Map correlation coefficients				
CC mask	0.74	0.78	0.73	0.6
CC box	0.65	——	——	——
CC peaks	0.65	——	——	——
CC volume	0.72	0.73	0.72	0.58

**Model geometry**	**Full model**	**Octamer**	**DNA**	**DDM1**
Ramachandran plot				
Outliers (%)	0	0	——	0
Allowed (%)	0.67	0	——	1.72
Favored (%)	99.33	100	——	98.28
Ramachandran plot Z-score				
Whole	−0.32 ± 0.23 (N = 1197)	0.18 ± 0.30 (N = 733)	——	−1.11 ± 0.36 (N = 464)
Helix	0.25 ± 0.19 (N = 681)	0.72 ± 0.23 (N = 492)	——	-0.96 ± 0.34 (N = 189)
Sheet	1.72 ± 0.73 (N = 55)	——	——	1.72 ± 0.73 (N = 55)
Loop	−1.05 ± 0.26 (N = 461)	−1.00 ± 0.36 (N = 241)	——	−1.10 ± 0.38 (N = 220)
CaBLAM outliers (%)	0.4	0.3	——	0.7
Rotamers				
Poor (%)	0.1	0	——	0.24
Favored (%)	98.28	98.72	——	97.62
R.M.S. deviations				
Bond lengths (Å)	0.004	0.004	0.004	0.004
Bond angles (°)	0.759	0.757	0.716	0.837
Geometry outliers				
C_α deviations (%)	0.08	0	——	0.22
C_β deviations	0	0	——	0
Bad bonds	0 / 9,894	0 / 5,991	0 / 6,484	0 / 3,903
Bad angles	0 / 13,296	0 / 8,040	0 / 10,003	0 / 5,256
Cis prolines	0 / 39	0 / 24	——	0 / 15
Chiral volume outliers	0 / 2633	0 / 931	0 / 1,128	0 / 574
Clashscore (all atoms)	3.22	1.97	2.79	5.57
MolProbity score	1.11	0.96	——	1.3
B-factors ($Å^2$) (min / max / mean)				
Protein	12.6 / 98.4 / 45.8	12.6 / 93.9 / 30.7	——	36.1 / 98.4 / 69.2
Nucleotide	28.84 / 201.26 / 87.65	——	28.8 / 201.3 / 87.7	——
